# Correction: Recent advances utilized in artificial switchable catalysis

**DOI:** 10.1039/d5ra90042e

**Published:** 2025-04-07

**Authors:** Arash Ghorbani-Choghamarani, Zahra Taherinia

**Affiliations:** a Department of Organic Chemistry, Faculty of Chemistry, Bu-Ali Sina University Hamedan 6517838683 Iran a.ghorbani@basu.ac.ir +98 8138380709 +98 8138282807; b Department of Chemistry, Ilam University P.O. Box 69315516 Ilam Iran z.taherinia@ilam.ac.ir

## Abstract

Correction for ‘Recent advances utilized in artificial switchable catalysis’ by Arash Ghorbani-Choghamarani, *et al.*, *RSC Adv.*, 2022, **12**, 23595–23617, https://doi.org/10.1039/D2RA03842K.

The authors regret text overlap with a number of different sources. Although many of the sources have been cited, some citations are missing and the text should have been rewritten to avoid the overlapping text. Additionally, although ref. 9 of the original article was cited in Section 1, it should also have been cited on page 23599 in Section 1.1.

The missing citations have been included in the reference list herein.^1–7^

The Royal Society of Chemistry apologises for these errors and any consequent inconvenience to authors and readers.

## References

[cit1] Mercer S. M., Robert T., Dixon D. V., Jessop P. G. (2012). Catal. Sci. Technol..

[cit2] Göstl R., Senf A., Hecht S. (2014). Chem. Soc. Rev..

[cit3] Harsha Vardhan Reddy K., Prakash Reddy V., Shankar J., Madhav B., Anil Kumar B. S. P., Nageswar Y. V. D. (2011). Tetrahedron Lett..

[cit4] Schrick M. P., Kabelo Ramollo G., Susanne Hirschbiegel C.-M., Fernandes M., Lemmerer A., Förster C., Bezuidenhout D. I., Heinze K. (2024). Organometallics.

[cit5] Blanco V., Leigh D. A., Marcos V. (2015). Chem. Soc. Rev..

[cit6] Choudhury J. (2018). Tetrahedron Lett..

[cit7] Vassalini I., Alessandri I. (2018). Catalysts.

